# Application of a Clapeyron-Type Equation to the Volume Phase Transition of Polymer Gels

**DOI:** 10.3390/gels6030025

**Published:** 2020-08-14

**Authors:** Toshikazu Takigawa, Jun-ichi Horinaka

**Affiliations:** Department of Material Chemistry, Kyoto University, Nishikyo-ku, Kyoto 615-8510, Japan; horinaka.junichi.5c@kyoto-u.ac.jp

**Keywords:** stimuli responsive gel, volume phase transition, Clapeyron equation, coefficient of performance, work efficiency, gel actuator, transition entropy, transition enthalpy

## Abstract

The applicability of the Clapeyron equation to the volume phase transition of cylindrical poly(*N*-isopropylacrylamide)-based gels under external force is reviewed. Firstly, the equilibrium conditions for the gels under tension are shown, and then we demonstrate that the Clapeyron equation can be applied to the volume phase transition of polymer gels to give the transition entropy or the transition enthalpy. The transition enthalpy at the volume phase transition obtained from the Clapeyron equation is compared with that from the calorimetry. A coefficient of performance, or work efficiency, for a gel actuator driven by the volume phase transition is also defined. How the work efficiency depends on applied force is shown based on a simple mechanical model. It is also shown that the force dependence of transition temperature is closely related to the efficiency curve. Experimental results are compared with the theoretical prediction.

## 1. Introduction

More than forty years have passed since the discovery of volume phase transition in actual polymer gels [1], and now the volume phase transition appears to be familiar for stimuli-responsive gels [1,2,3,4,5,6,7,8,9,10,11,12,13,14,15,16]. The phase transition occurs in a macroscopic scale, and is thus the matter of thermodynamics [1,2,3,4,5,6,7,8,9,10,11,12], so this is often analyzed and discussed with the analogy of the phase transition of van der Waals fluids [3,11,12], and thus “volume phase transition” was basically used to mean a discontinuous change in volume (*V*) upon an infinitesimal change in a control variable [1,2,3,4,5,6,7,8,9,10,11,12]. For thermo-sensitive gels, temperature (*T*) is used as the control variable. In principle, the volume phase transition belongs to the category of first-order phase transition. There exist some cases that the plot of *V* against *T* becomes continuous. Even in this situation, if the slope of curves (∂*V*/∂*T*) diverges at a certain temperature, then the system is called to show the second-order phase transition [8,9]. When the slope of the curves is not divergent, the curves show only a continuous change with a finite slope over an entire region. The use of “transition” appeared to be avoided for these curves. In the early stage of research on the volume phase transition, the attention was paid to the physical aspect of the phase transition [1,2,3,4,5,6,7,8,9,10,11,12], but then the research spread over various fields of polymer science [13,14,15] and engineering, especially in biomedical applications [16,17,18,19,20,21,22]. During this extending process, the meaning of the word “transition” has changed such that the word is now used in a broader sense and thus confusedly [14,15,21,22]. In this paper, we use the word “transition” in the original meaning, in principle, although curious behavior such as the first-order phase transition has been observed for the volume phase transition [8,9]. 

When the phase transition occurs in the first-order manner, two gel phases coexist at the transition point. Actually, it is well known that poly(*N*-isopropylacrylamide) (PNIPA)-based polymer gels undergo the volume phase transition, and that the coexistence state clearly emerges for cylindrical gel specimens [3,5,6,7,8,9,10,11,12,13,14,15,16,17,18,19,20,21,22]. Interestingly, this coexistence is observed for long cylindrical gels but is not for gels in the other geometries [5,8,9,10]. The long cylinder might be one of the ideal geometries for the volume phase transition of polymer gels. Why the coexistence is limited to the specific geometry probably comes from the fact that the volume phase transition is a phenomenon of solids, not fluids [11]. For the phase transition of solids, the interface becomes rather thick and thus the effect of the interface cannot be ignored. This may cause the curious behavior as the first-order phase transition; for example, the Gibbs phase rule [23] is broken at the volume phase transition of gels [8,9]. Thus, the fact that the volume phase transition is the phenomenon of solid also becomes important. 

There are similarities between the volume phase transition of polymer gels and the liquid-gas phase transition of the van der Waals fluids, but there also exist marked differences. For example, the former is the phase transition of solids and the latter is that of fluids, as stated previously. In addition, the surrounding solvent is indispensable to the volume phase transition [2,3,4,5,6,7,8,9,10,24], which means that the phase structure is different between these systems. For the van der Waals fluids, *V* can be written as a function of *T* and pressure (*p*), namely *V* = *V*(*T*, *p*) [23]. We can choose two variables, *T* and *p*. If we settle *T* in advance, then *V* becomes a function of *p* at a fixed *T*. The transition state is the two phase-one component system, and thus for the variance in the Gibbs phase rule (*F*), we have *F* = 1 at the transition point, indicating that the pressure at the phase transition is automatically settled (by the Maxwell construction) [6,23]. For electrically neutral gels, on the other hand, *V* can be written as a function of *T* and the osmotic pressure (*π*), because the gels always coexist with the outside solvent: *V* = *V* (*T*, *π*). The equilibration condition *π* = 0 consumes a degree of freedom in *F*. Because the coexistence state at the phase transition is the two component-three phase system and thus *F* = 1 at the transition point, which is eventually identical to that for the van der Waals fluids but is quite apparent. No degree of freedom is also left for the transition temperature for the volume phase transition [8,9]. In the Tanaka theory [3,25], which is made up to describe the volume phase transition of ionic gels based on the Flory–Huggins expression for the osmotic pressure [26,27,28], the number density of counter ions (*ϕ*_ion_) is introduced as an additional degree of freedom: In the *V*-*T* curves of the gels *ϕ*_ion_ acts as *T* in the *P*-*V* curves of the van der Waals fluids. However, this control variable becomes meaningful only when various gels differing in *ϕ*_ion_ are prepared and examined. Potentially, there exist several candidates of the additional control variables other than *ϕ*_ion_, and one of them is the mechanical force (*f*; positive for tension). It is reported for the PNIPA-based gels in solvent that *f* actually works as a control variable under *π* = 0 [8,9,10,12,29,30]. This comes from the fact that gels are solid and thus the mechanical force is applicable. It is important to recall that the volume phase transition occurs three-dimensionally or isotropically in principle while the deformation by external force occurs anisotropically. This difference affects the phase transition behavior of gels under tension. 

In this mini-review, applicability of the Clapeyron equation to the volume phase transition of PNIPA-based gels under external force (***f*** as a vectorial quantity and *f* = |***f***|) is reviewed, although this may be limited to the cylindrical geometry at present. Firstly, the equilibrium conditions for the gels under tension are shown and why the *f* dependence curve of transition temperature builds up the phase boundary is also shown. Then, we demonstrate that the Clapeyron equation, which is the prototype of the Clausius–Clapeyron equation [23], is applicable to the volume phase transition of polymer gels and gives the transition entropy (Δ*S*) or the transition enthalpy (Δ*H*). Although Δ*H* at the volume phase transition has been obtained by calorimetry [7,12,31,32,33,34], the Clapeyron equation offers a new method to estimate Δ*H* at the phase transition. Finally, a coefficient of performance (i.e., work efficiency) for gel actuator driven by the volume phase transition (*c*) is defined and how *c* changes with *f* is discussed based on the rubber elasticity theory [35,36]. The PNIPA-based polymer gels were intended to apply to a soft actuator. Although this application is now recognized to be non-realistic mainly due to slow response speed [37,38], we think that *c* is a very important parameter because *c* determines how the transition temperature moves with *f*. In the final section, comparison with experimental data is made.

## 2. Equilibrium Conditions for Coexistence of Two Gel Phases in Solvent 

**Figure 1:**
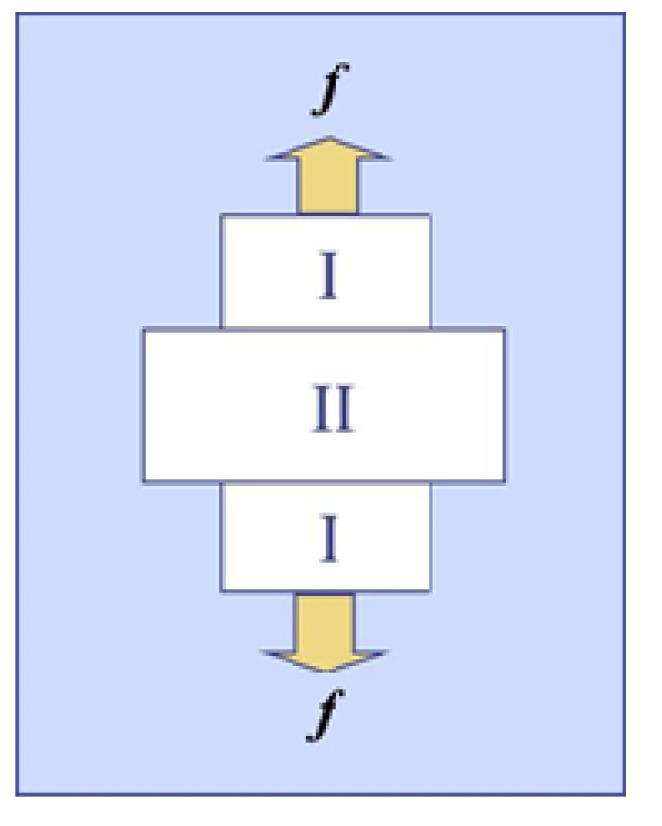
Cylindrical polymer gel under tension at the transition point. Two gel phases (I and II) and an outside solvent coexist. The upper and lower regions specified by I are the same.

Suppose that polymer gel is electrically neutral and are made up by chemical crosslinks. When the gel coexists with the outside solvent under tension, the free energy (*G*) of the gel is defined by *G* = *U* − *TS* − ***f****⋅**l***, where *U*, *S* and ***l*** are the internal energy, the entropy, and the position vector, respectively. If *G* is measured from a reference state where the polymer and the solvent are isolated, then *G* can be written as *G* = *n*_p_(*μ*_p_ − *μ*_p_^0^) + *n*_s_(*μ*_s_ − *μ*_s_^0^), where *n*_p_ and *n*_s_ are respectively the numbers of polymer strand and the solvent molecules in the gel, and *μ*_p_ and *μ*_s_ are respectively the chemical potentials of polymer strand and solvent, *μ*_p_^0^ and *μ*_s_^0^ being those of polymer strand in the pure network and of the pure solvent, respectively. It should be noticed here that *n*_p_ is kept constant. Figure 1 schematically shows the coexistence state for a cylindrical gel in solvent under tension, where the two gel-gel interfaces are assumed be so thin that the free energy of the interfaces is negligible. For the cylindrical gels, the coexistence emerges in ABA morphology probably due to an end effect of the geometry. Both A domains at the ends are believed to be identical, and thus the coexistence state in the gel stays at a tri-phasic equilibrium because the pure solvent phase additionally exists outside the gel. We designate these two gel phases as Phase I and Phase II, as shown in the figure. Because no interfacial energy exists by the assumption, *G* in the coexistence state can be written as [24]

(1) $$G\left(T,\;f,\;n_{p}^{I},\;n_{p}^{II}\right)=\left(\mu_{p}^{I}-\mu_{p}^{0}\right)n_{p}^{I}+\left(\mu_{p}^{II}-\mu_{p}^{0}\right)n_{p}^{II}+\left(\mu_{s}^{I}-\mu_{s}^{0}\right)n_{s}^{I}+\left(\mu_{s}^{II}-\mu_{s}^{0}\right)n_{s}^{II}$$

where the superscripts I and II stand for Phases I and II, respectively. On heating, Phase I corresponds to the collapsed phase and Phase II to the swollen phase, but the situation is inverted on cooling. Because the variation of *G* (δ*G*) given by

(2) $$\delta G=\left(\mu_{p}^{I}-\mu_{p}^{\mathrm{II}}\right)\delta n_{p}^{I}+\left(\mu_{s}^{I}-\mu_{s}^{0}\right)\delta n_{s}^{I}+\left(\mu_{s}^{II}-\mu_{s}^{0}\right)\delta n_{s}^{II}$$

must be zero at equilibrium, we have

(3a) $$\mu_{s}^{I}=\mu_{s}^{II}=\mu_{s}^{0}$$

(3b) $$\mu_{p}^{I}=\mu_{p}^{\mathrm{II}}$$

where δ(*n*_p_^I^ + *n*_p_^II^) = 0 is used [24]. Equation (3a) expresses the condition for the chemical potential for solvent, and is satisfied as long as the equilibrium swelling is attained. Equation (3b) determines the coexistence condition for the networks in the gel phases. 

### 2.1. Clapeyron Equation

By applying Equation (3b) to the point (*T*, ***f***) and the other point in the vicinity (*T* + d*T*, ***f*** + d***f***), we have [24]

(4a) $$\frac{\partial \left(\mu_{p}^{I}-\mu_{p}^{\mathrm{II}}\right)}{\partial T}dT+\frac{\partial \left(\mu_{p}^{I}-\mu_{p}^{\mathrm{II}}\right)}{\partial f}\cdot df=0$$

**Figure 2:**
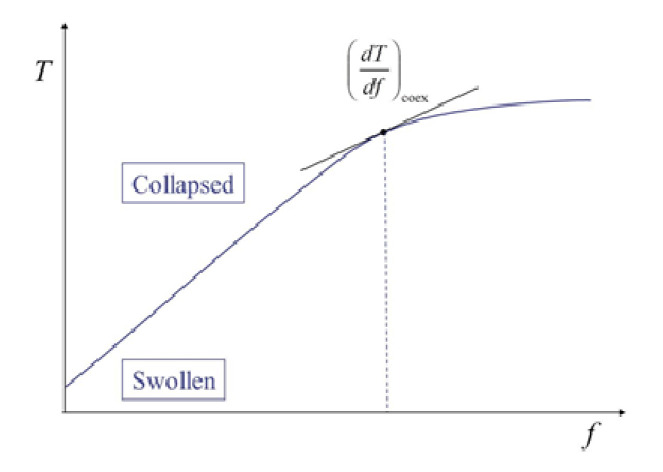
Schematic representation of phase diagram on the force (*f*)-temperature (*T*) plane. PNIPA based polymer gels show this type of phase diagram. The curve in the figure is the phase boundary and also corresponds to the force dependence of the transition temperature.

The physical meaning of Equation (4a) is not so clear as it is because ***f*** and ***l*** are basically defined for the bulk gel, but this can also be written as [24]

(4b) $$\frac{\partial \Delta G}{\partial T}dT+\frac{\partial \Delta G}{\partial f}\cdot df=0$$

if we recall that *G* is given by Δ*G* = *G*^I^ − *G*^II^, and *G*^I^ = *n*_p_(*μ*_p_^I^ − *μ*_p_^0^) and *G*^II^ = *n*_p_(*μ*_p_^II^ − *μ*_p_^0^) at swelling equilibrium (see Equation (1)). Thus, we have

(4c) ΔSdT+Δl⋅df=0

where Δ*S* = *S*^I^ − *S*^II^ and Δ***l*** = ***l***^I^ − ***l***^II^ because (∂*G^i^*/∂*T*) = −*S^i^* and (∂*G^i^*/∂***f***) = −***l****^i^* (*i* = I, II) [24]. Equations (4a)–(4c) can be applied to ionic gels. Rearranging Equation (4c) leads to

$${\left(\frac{dT}{df}\right)}_{\mathrm{coex}}=-\frac{\Delta l}{\Delta S}$$

(5) $$\;\;\;\;=-\frac{T\Delta l}{\Delta H}$$

where d*f* = |d***f***| and Δ*l* = |Δ***l***| [24]. The quantity Δ*H* in Equation (5) stands for the change in enthalpy by the phase transition given by Δ*H* = *T*Δ*S*. The subscript “coex” represents that the derivative should be taken for the coexistence curve (i.e., phase boundary). Figure 2 schematically shows a phase diagram of a gel showing the volume phase transition. It is important to notice that the phase boundary corresponds to the *f*-dependence curve of the transition temperature. Once we obtain the phase diagram and know Δ*l* at a given *f*, then we have Δ*H* from Equation (5). To compare the above transition enthalpy with that obtained by other methods, Δ*H* at *f* = 0 (Δ*H*_0_) given below is used.

(6) $$\Delta H_{0}=-T_{0}\underset{f\to 0}{\lim}\Delta l\left(f\right){\left(\frac{dT}{df}\right)}_{\mathrm{coex}}^{-1}$$

Here, *T*_0_ is the transition temperature at *f* = 0. The critical point for the volume phase transition under tension corresponds to the point at Δ*l* = 0 on the boundary. When the discontinuity in length remains even at the zero-force state (now we assume this situation), the critical point emerges under compression (i.e., negative *f* state) in principle because Δ*l* is an increasing function of *f* in the small *f* region, as will be shown later. 

When the deformation by the external force ***f*** is small enough compared with that by the phase transition, Equation (5) can be formally transformed into the conventional Clapeyron equation [12]. Letting *p* be an “average” pressure, *p* can be defined *p* = −Tr(***σ***)/3 with the stress tensor ***σ*** [39]. Here, Tr***A*** stands for the sum of diagonal elements of the matrix ***A***. For the uniaxial deformation, *p* is simply given by *p* = −*σ*/3 with the elongational stress acting on the gel (*σ*). If we introduce the gel volume (*V*) and the volume change by the phase transition (Δ*V*), then *f*Δ*l* can be formally replaced by −*p*Δ*V* because Δ*V* ≅ 3*A*Δ*l* and *p* = −*σ*/3 = −*f*/3*A* with the force-acting area just before transition (*A*). In this case, we have the following conventional form [12]:

(7) $${\left(\frac{dp}{dT}\right)}_{\mathrm{coes}}=\frac{\Delta S}{\Delta V}=\frac{\Delta H}{T\Delta V}$$

It should be noticed that the above transformation of variables is just formal. Actually, what is kept constant during phase transition is not *f,* but *p*. 

### 2.2. Efficiency of Work at the Volume Phase Transition

**Figure 3:**
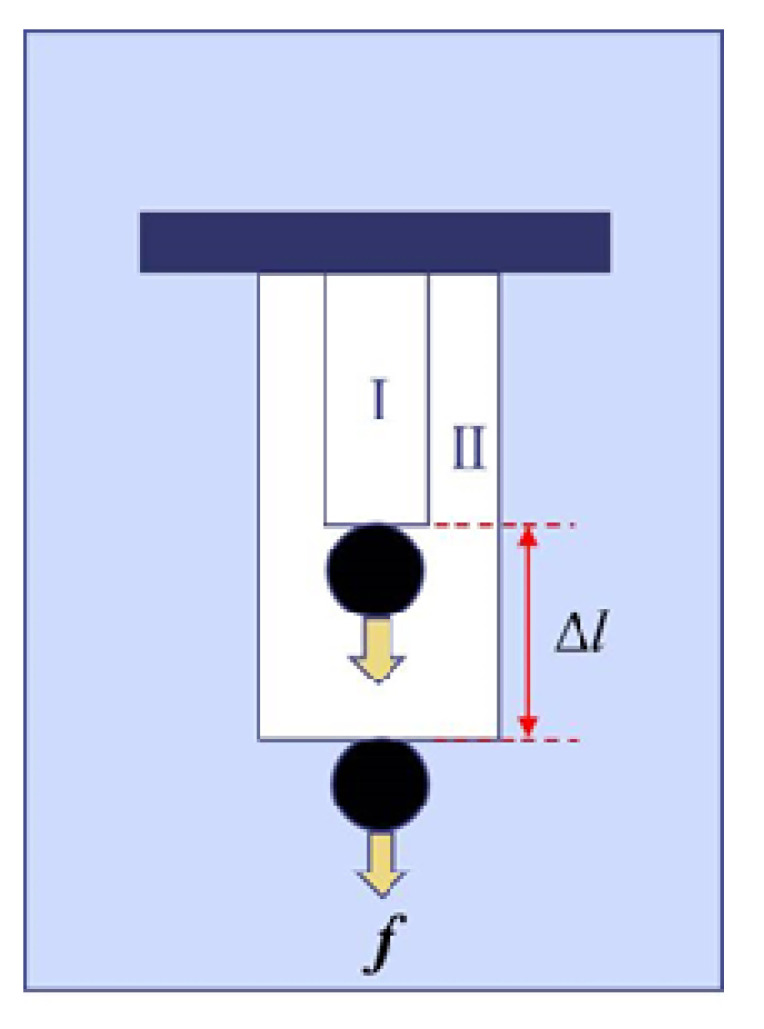
Work done by a cylindrical polymer gel at the transition. Weight (*f* in force) is lifted up by Δ*l* through the collapsing process. Phase I and Phase II correspond to collapsed and swollen phases, respectively, as is the case of Figure 1.

Figure 3 schematically shows the work at the phase transition from Phase I to Phase II of the gel under a constant force ***f***. Here, we define *c* by [24]

$$c\equiv -\frac{\Delta W}{\Delta H}$$

(8) $$\;\;\;=-\frac{f\Delta l}{\Delta H}$$

where −Δ*W* is the work by gel and in our case Δ*W* = *f*Δ*l*. By combining Equations (5) and (8), we have [24]

(9) $$c=\frac{f}{T}{\left(\frac{dT}{df}\right)}_{\mathrm{coes}}={\left(\frac{d\ln T}{d\ln f}\right)}_{\mathrm{coes}}$$

If *c* depends only on *f* (i.e., *c* = *c*(*f*)), Equation (9) gives [40]

(10a) $$T\left(f\right)=T_{0}\exp\left(\int_{\;0}^{\;f}\frac{c\left(f^{\prime }\right)}{f^{\prime }}df^{\prime }\right)$$

This determines the phase boundary in the phase diagram to which the Clapeyron equation is applicable. When (*T* − *T*_0_)/*T*_0_ << 1, which must be satisfied in most cases, *T* is expressed by [40]

(10b) $$T\left(f\right)≅T_{0}\left\{1+\int_{\;0}^{\;f}\frac{c\left(f^{\prime }\right)}{f^{\prime }}df^{\prime }\right\}$$

## 3. A Mechanical Model for the Volume Phase Transition 

**Figure 4:**
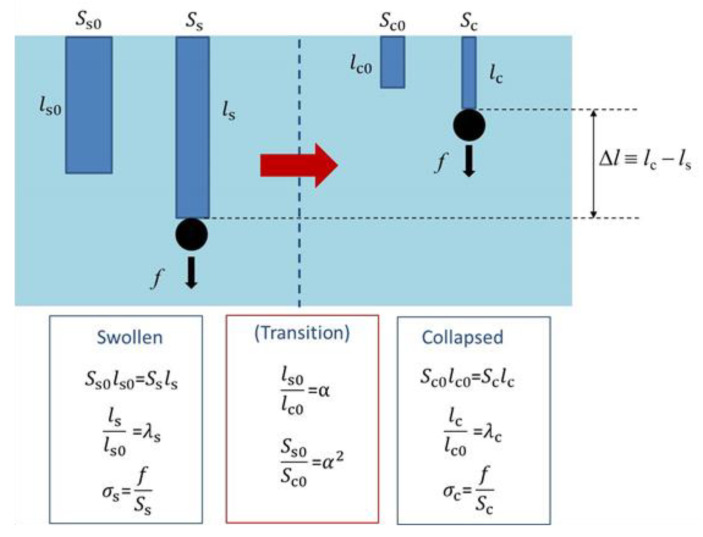
Schematic representation of the change in dimension of a polymer gel by loading as well as by transition. For details, see text.

Here, we consider a mechanical model for the volume phase transition of cylindrical polymer gel [40]. Firstly, we designate the length and the cross-sectional area without tension in the collapsed state as *l*_c0_ and *A*_c0_, respectively. Similarly, let *l*_s0_ and *A*_s0_ be the length and the cross-sectional area without tension in the swollen state, respectively. Because the volume phase transition without tension occurs isotropically, we set

(11) $$\alpha =\frac{l_{s0}}{l_{c0}},\;\alpha^{2}=\frac{A_{s0}}{A_{c0}}$$

where *α* is the linear swelling ratio at the phase transition and thus *α* > 1. These are depicted in Figure 4. Note that the volume in the collapsed state without tension (*V*_c0_) is given by *V*_c0_ = *l*_c0_*A*_c0_ and that in the swollen state without tension (*V*_s0_) is given by *V*_s0_ = *l*_s0_*A*_s0_, and thus *V*_s0_/*V*_c0_ = *α*^3^. When tension (*f* in force) is applied to the gel, the gel is stretched. We describe this deformation (uniaxial elongation) by using the stretch ratio as follows.

(12) $$\lambda_{c}=\frac{l_{c}}{l_{c0}},\;\lambda_{s}=\frac{l_{s}}{l_{s0}}$$

Here, *l*_c_ and *l*_s_ are the lengths in the collapsed and swollen states, respectively, and *λ*_c_ and *λ*s are the stretch ratios in the collapsed and swollen states, respectively. For simplicity, we assume here the incompressibility for the uniaxial elongation in the both states, which gives *l*_c0_*A*_c0_ = *l*_c_*A*_c_ and *l*_s0_*A*_s0_ = *l*_s_*A*_s_ with the cross-sectional area in the collapsed state (*A*_c_) and that in the swollen state (*A*_s_). It is noticed that the incompressibility ignores the effects of tension-induced re-swelling of polymer gels [41,42]. The true stresses in the collapsed and swollen states (*σ*_c_ and *σ*_s_, respectively) are given by

(13) $$\sigma_{c}=\frac{f}{A_{c0}},\;\sigma_{s}=\frac{f}{A_{s0}}$$

If we assume that *σ*_c_ and *σ*_s_ can be expressed by the classical theory of rubber elasticity [35,36], then we have

(14) $$\sigma_{c}=G_{c}\left(\lambda_{c}^{2}-\lambda_{c}^{-1}\right),\;\sigma_{s}=G_{s}\left(\lambda_{s}^{2}-\lambda_{s}^{-1}\right)$$

where *G*_c_ and *G*_s_ are the moduli in the collapsed and swollen states, respectively. For chemical gels *G*_c_ and *G*_s_ are known to be related as *G*_c_/*G*_s_ = *α* because the number of active chains is kept constant before and after phase transition [28,35,36], but we set here the ratio *G*_c_/*G*_s_ to be just a numerical constant *r* (i.e., *r* = *G*_c_/*G*_s_) because we know that the physical crosslinks are introduced by the collapsing transition [42,43]. Finally, from Equation (14), we have

(15) $$r\left(\lambda_{c}-\frac{1}{\lambda_{c}^{2}}\right)=\alpha^{2}\left(\lambda_{s}-\frac{1}{\lambda_{s}^{2}}\right)$$

and for the change in length by the phase transition Δ*l* defined by Δ*l* ≡ *l*_c_ − *l*_s_ is written as

(16) $$\Delta l=l_{s0}\left(\frac{\lambda_{c}}{\alpha }-\lambda_{s}\right)$$

These give the exact solution of *c* as a function of *λ*_s_ and also the expression for *T*, but here we examine how *c* and *T* vary with *f* with a simpler method.

When *f* is small, we can expect that *λ*_s_ ≅ 1 and *λ*_c_ ≅ 1 (Equation (12)). This gives Δ*l* ≅ *l*_s0_{(1/*α*) − 1}; thus, Δ*l* becomes a negative constant because *α* > 1. In this *f* region, Δ*H* ≅ Δ*H*_0_ (> 0) is also expected. Therefore, *c* > 0 and *c* ∝ *f* (Equation (8)). On the other hand, in the large *f* region where *λ*_s_ >>1 and also *λ*_c_ >>1, *f* ∝ *λ*_s_. It is also shown that *λ*_c_ ≅ (*α*^2^/*r*)*λ*_s_ (Equation (15)) and Δ*l* ≅ *l*_s0_{(*α*/*r*) − 1}*λ*_s_ (Equation (16)). Thus, *c* behaves as *c* ∝ *f*^2^ if Δ*H* remains constant also in this *f* region. These two asymptotic relations in both small and large *f* regions could give the following expression for *c* in the whole region of *f*.

(17) $$c=af^{2}+bf$$

**Figure 5:**
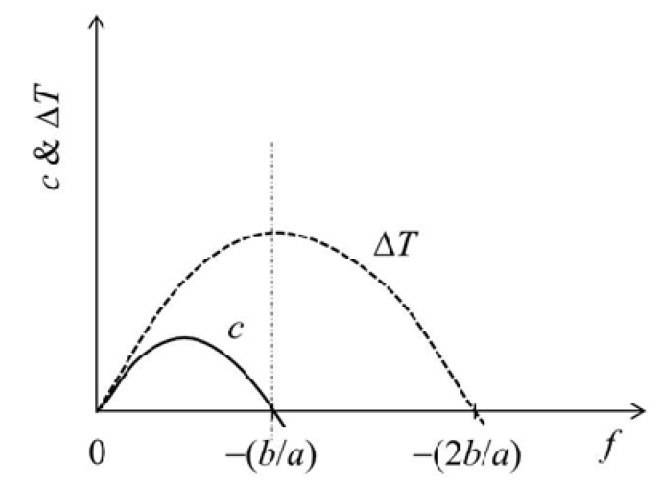
Expected force (*f*) dependence of the coefficient of performance (*c*) and the increment of the transition temperature (Δ*T*).

**Figure 6:**
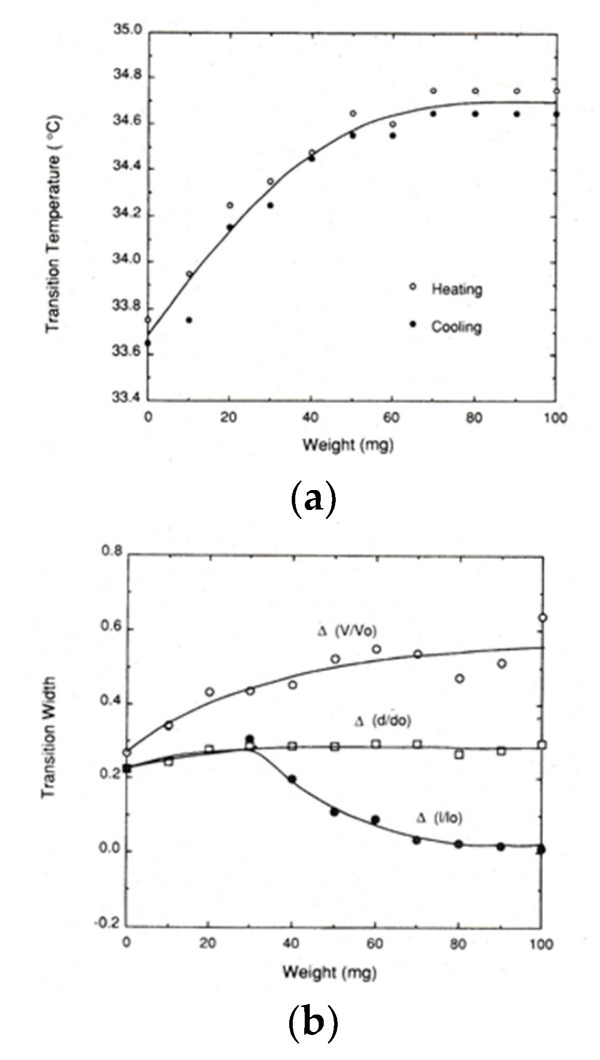
Uniaxial stress dependence of (**a**) the transition temperature and (**b**) the transition widths (*d*/*d*_0_, *l*/*l*_0_ and *V*/*V*_0_) for PNIPA gels [10]. For details, see text.

Here, *a* and *b* are numerical constants. For *b* we know that *b* > 0 because *b* ≅ *l*_s0_{1 − (1/*α*)}*λ*_s_/Δ*H*_0_. Equations (10b) and (17) also give

(18) $$\frac{\Delta T}{T_{0}}=\frac{a}{2}f^{2}+bf$$

where Δ*T* = *T* − *T*_0_. This determines the *f* dependence of *T*, but it should be recalled again that this curve corresponds to the phase boundary between swollen and collapsed phases on the phase diagram. When the physical crosslinks are introduced by the collapsing transition, the modulus in the collapsed state is enhanced. This means that *r* > *α*, giving that Δ*l* < 0 and *a* > 0 in the large *f* region because Δ*l* ≅ *l*_s0_{(*α*/*r*) − 1}*λ*_s_ and the sign of *a* becomes identical to that of {1 − (*α*/*r*)} if Δ*H* > 0. However, the transition temperature vs *f* plots for real gels are convex in shape (as will be shown later in Figure 6a in the next section, for example). This suggests that *a* < 0 (see Equation (18)) for the real gels. Although we do not know exactly why *a* becomes negative, the inequality between *r* and *α* is essentially determined by the properties at small *f* but *a* is affected basically by the properties at large *f*. Thus, in the collapsed state a marked strain softening at large *f* may occur to give the negative *a*. The convex curve also suggests that Δ*l* > 0 in the large *f* region. The *f* dependence curves of *c* and Δ*T* for *a* < 0 (Equations (17) and (18), respectively) are schematically shown in Figure 5. For the *c* curve, *c* ≥ 0 for 0 ≤ *f* ≤ −*b*/*a* and *c* < 0 for *f* > −*b*/*a*, while Δ*T* increases with increasing *f* to show a maximum at *f* = −*b*/*a* where *c* = 0, and then starts to decrease. The negative *c* occurs because a reduction in cross-sectional area becomes dominant in this *f* region.

## 4. Comparison with Experiments

To our knowledge, the first report on the experiment of how external force affects the volume phase transition behavior of gels was made by Hirotsu and Onuki [29]. It was shown that the transition temperature increases with increasing tension for PNIPA gel by experiment [29]. They also showed that the shift of transition temperature by applied tension can be explained by a Flory-type free energy if a concentration dependent interaction parameter between polymer and solvent (*χ*) is introduced. Hereafter, we call the free energy expression the Hirotsu-Onuki (HO) model [29]. A more detailed experiment under load was also made by Suzuki [10]. In Figure 6a his data on force-transition temperature relation for lightly crosslinked PNIPA gels (1BIS gel according to his notation) are shown. Although a hysteresis exists between the data on heating and cooling, the transition temperature increases with increasing applied force (or weight, also *f*), but in the large force region, saturation behavior emerges. He also found that the shift factor by force can be well explained by the HO model at small *f* but the saturation behavior at large *f* cannot be explained by the HO model [10]. Concerning the shape of curve, the curve is not exactly parabolic but remains globally convex, as suggested by Equation (18).

Figure 6b shows the transition widths for the PNIPA gels as a function of applied force [10]. Here, *d*, *l* and *V* are the diameter, length, and volume of the cylindrical gel, respectively, and the subscript 0 stands for the value just after preparation. The quantities, Δ(*d*/*d*_0_), Δ(*l*/*l*_0_) and Δ(*V*/*V*_0_) are the transition widths of the normalized diameter *d*/*d*_0_, normalized length *l*/*l*_0_ and normalized volume *V*/*V*_0_, respectively [10]. As can be seen from the figure, Δ(*d*/*d*_0_) increases with increasing *f* and then levels off at about *f* = 40 mg. For the Δ(*l*/*l*_0_) curve, Δ(*l*/*l*_0_) moves on the same path as Δ(*d*/*d*_0_) in the region of *f* < 40 mg. This is because the effect of *f* is negligible and thus the phase transition occurs almost isotropically in the small *f* region. This means that in the small *f* region we can apply Equation (7) to estimate the transition enthalpy. At about *f* = 40 mg, Δ(*l*/*l*_0_) turns to decrease and then moves to zero, suggesting that the dimensional change at the phase transition becomes restricted in the loaded direction as *f* increases. In addition, it is also reported for the other PNIPA-based gel systems that the transition temperature tends to decrease, rather than levels off, with increasing *f* at very large deformations, leading to negative *c* [30]. Concerning the change in volume, Δ(*V*/*V*_0_) monotonously increases with increasing *f*, as is clear from the figure.

**Figure 7:**
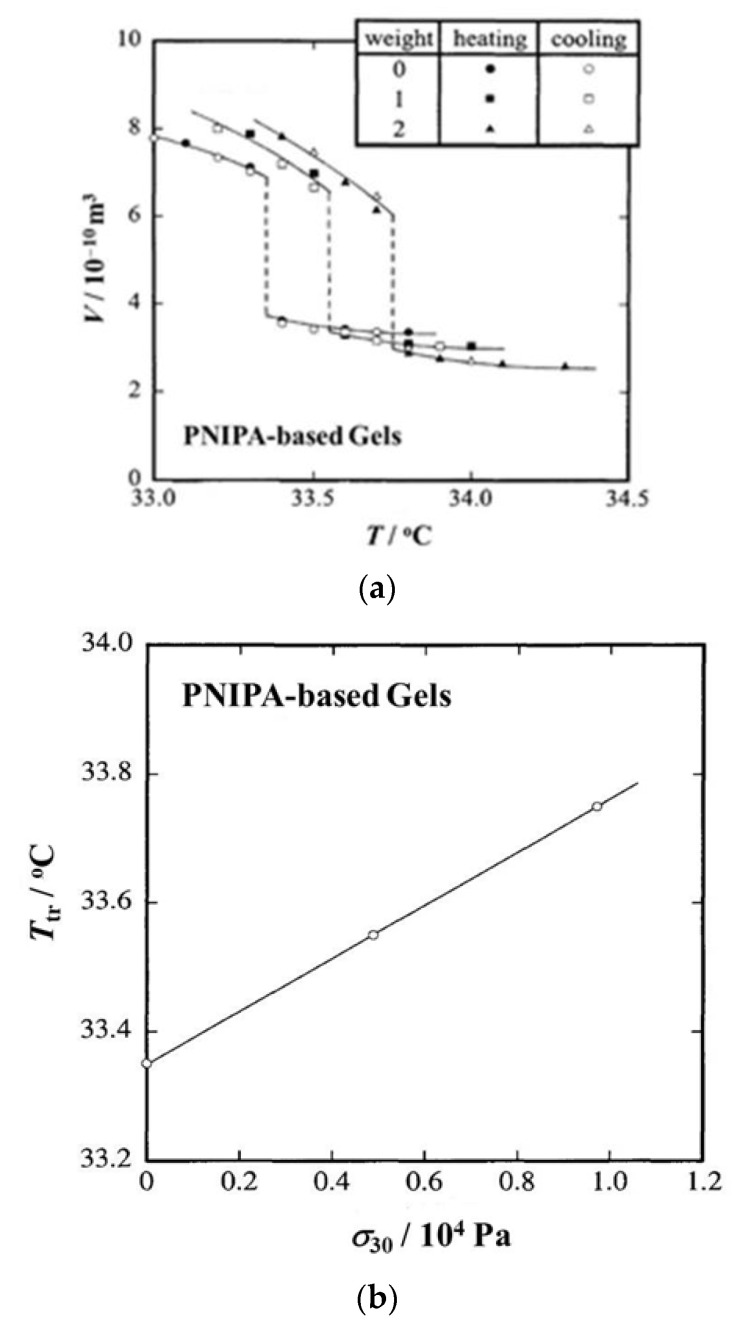
(**a**) Temperature dependence of gel volume and (**b**) stress dependence of transition temperature for PNIPA based gel [12]. Here, *σ*_30_ stands for the true stress at 30 °C.

Figure 7a shows *T* dependence curves of *V* for a *N*-isopropylacrylamide and sodium acrylate copolymer hydrogel in water [12]. Here, spherical metal weights (35 mg/each) were used for loading and thus numerals in the “weight” column of the figure stand for the number of metal weights used. In any cases, the discontinuous volume change, or volume phase transition, occurs, and the degree of discontinuity remains almost constant. On the other hand, the transition temperature moves to the higher temperature side as the number of weights (namely, *f*) increases. In Figure 7b, the transition temperature (*T*_tr_ in the figure) is plotted against the true stress at 30 °C (*σ*_30_), which was used as the pressure measure instead of *σ* just before phase transition [12]. The three data points fall on a single line and *T*_tr_ linearly increases with increasing *σ*_30_. The line in the figure corresponds to the phase boundary between collapsed (above the line) and swollen (below the line) phases, and the slope of the line gives Δ*H* for the volume phase transition (Equation (7) with *p* = −*σ*_30_/3). Table 1 summarizes *T*_tr_, volume change at the phase transition (Δ*V*) and transition enthalpy (Δ*H*) at three different values of *f* (in the number of weights). Here, Δ*V* and Δ*H* are shown as the quantities per unit mass of polymer network. As can be seen from the table, *T*_tr_ and Δ*V* remain constant regardless of *f* and Δ*H* is also kept almost constant. In Table 2, the transition enthalpy per unit mass of polymer network by DSC (Δ*H*_DSC_) and the transition temperature determined with the onset point of the peak on the DSC thermogram (*T*_DSC_) are listed together with the heating rate (*v*). Concerning *T*_DSC_, the values slightly increase with increasing *v* and are higher than *T*_tr_ by 2~4 °C. On the other hand, Δ*H*_DSC_ remains around 10 Jg^−1^ regardless of *v*, which is much larger than Δ*H*. The difference in transition enthalpy comes from the effect of phase separation, or dehydration of PNIPA chains, inside gels [31,32,33,34]. As is well known, PNIPA aqueous solutions become opaque when the systems are heated up to high temperatures. This corresponds to the phase separation originating from the fact that the PNIPA-water system has a lowest critical solution temperature (LCST)-type phase diagram [44,45,46,47]. Although opaqueness or phase separation also occurs in the PNIPA gels, the opaqueness completely disappears at equilibrium [8,9,48,49]. This equilibration, however, usually takes very long time, so that the DSC measurements inevitably detect the phase separation while the estimation by the Clapeyron equation does not contain the effect of phase separation: Only the method using the Clapeyron equation can estimate the transition enthalpy for the volume phase transition. It should be noticed that the opaqueness did not accompany the change in shape in the above study [12], but an interesting pattern formation may occur when the volume change is large and/or the shrinking speed is high [50].

**Figure 8:**
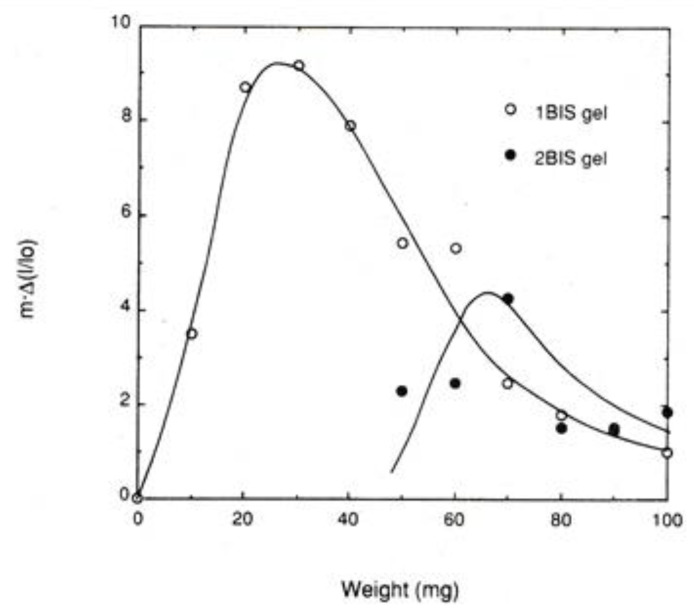
Mechanical work per unit length of PNIPA gels at the transition temperature on heating as a function of applied weight [10].

In Figure 8, normalized mechanical work at the transition temperature *m*⋅Δ(*l*/*l*_0_) is plotted against weight (or tension *f*) for two series of PNIPA gels differing in crosslink density, where the crosslink density of 2BIS is twice as high as that of 1BIS [10]. Here, *m* stands for the weight applied to the gel specimens. It should be noticed that these curves result in the *f* dependence curve of *c* defined in the previous section when Δ*H* stays constant. In both series, the normalized work curve shows a maximum. For 1BIS the curve is directly comparable to the data in Figure 6a,b, and the comparison shows that the *f* value at the peak for 1BIS curve, 30 mg, becomes identical to that at the inflection point for the Δ(*l*/*l*_0_) curve in Figure 6b. Concerning the transition temperature vs. *f* curve in Figure 6a, the slope of the curve decreases with increasing *f* and the value of slope finally becomes zero at about *f* = 60 mg. The *f* value is twice as large as the *f* value at the peak of the work curve. This must be compared with the relation between Equations (17) and (18), which is also shown in Figure 5. For the 2BIS curve, we have no comparable data, as is the case of 1BIS, but the 2BIS curve also shows a maximum.

**Figure 9:**
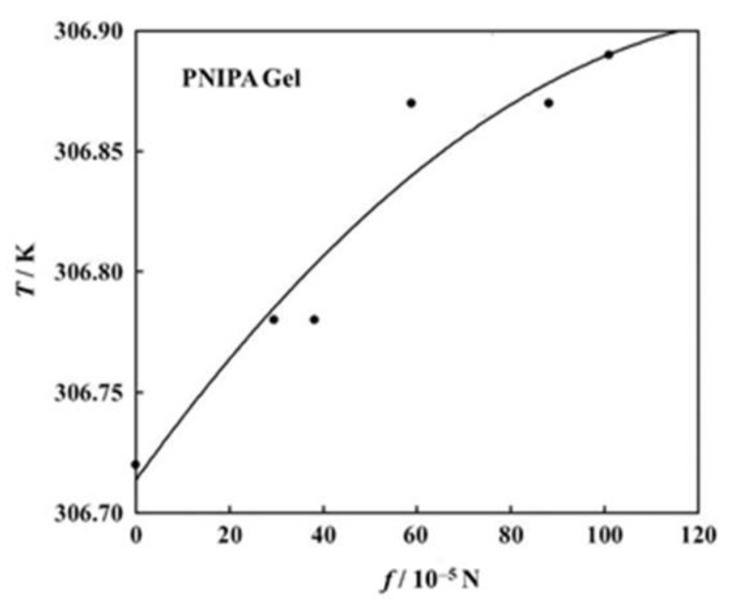
Force dependence of transition temperature for the PNIPA gel [40].

**Figure 10:**
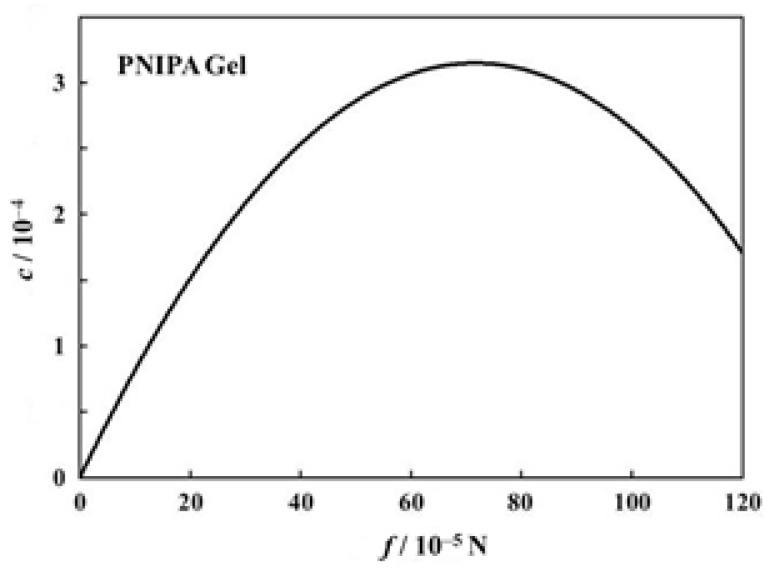
Coefficient of performance of the PNIPA gel as a function of force [40].

We try to estimate here the *c* values for the volume phase transition of PNIPA gels under tension. Firstly, another *f* dependence curve of transition temperature than that in Figure 6a is shown in Figure 9 [40]. Here, each symbol stands for the transition temperature determined as an average of on-heating and on-cooling measurements. The transition temperature shows a monotonous increase in this region of *f*. The solid curve in the figure stands for the best fit parabola (assuming the form in Equation (18)) for the data points, and the parameters decided were *a* = −6.144 × 10^2^ and *b* = 1.432 × 10^−3^ [40]. With these values we can draw the *f* dependence curve of *c* and thus obtained curve is shown in Figure 10. The *c* curve shows a maximum in this region of *f*, and the value at the maximum lies at most 3 × 10^−4^. We do not know whether this value is appropriate or not, because we have no comparable data for *c*. The fact that the *c* curve has a maximum appears to be consistent with the result shown in Figure 8. The emergence of maximum has also been reported for the stress-dependent curve of normalized work for poly(vinyl alcohol)/poly(acrylic acid) copolymer gels undergoing a pH-jump [51].

Until now, the definition and application of the Clapeyron equation for the volume phase transition of polymer gels under applied force were shown, where the discussion was basically limited to the gels in the cylindrical geometry. This is because the cylindrical geometry was ideal, and thus the coexistence of two gel phases was realized at the transition point. However, Hirotsu has shown that the temperature range where the coexistence is realized becomes larger than several degrees Celsius for ionic gels [8,9]. This is quite strange as the first-order phase transition, but is more serious because the transition temperature cannot be determined uniquely and thus the applicability of the Clapeyron equation is vanished. For the volume phase transition of cylindrical gels, swelling or collapsing initiates at the ends, probably due to an end effect originating from the fact that the end zone is different in circumstance from other parts. The interface between two gel phases is not so thin because the gels are solid, meaning that the elastic energy at the interface cannot be ignored. Therefore, the phase transition completes when the interface “melts” and this may require several degrees celsius for the ionic gels. Thus, the applicability of the Clapeyron equation is rather limited, but the Clapeyron equation works as a powerful tool as long as the temperature range for coexistence is not so large.

## Figures and Tables

**Table 1:** Force in the number of metal weight (*f*), transition temperature (*T*_tr_), volume change of gel specimen per unit mass of polymer network (Δ*V*) and transition enthalpy per unit mass of polymer network (Δ*H*).

*f*	*T*_tr_/°C	Δ*V*/10^−6^m^3^g^−1^	Δ*H*/Jg^−1^
0	33.35	−1.08	2.7
1	33.55	−1.06	2.6
2	33.75	−1.05	2.6

**Table 2:** Heating rate (*v*), transition temperature determined by DSC (*T*_DSC_) and transition enthalpy per unit mass of polymer network determined by DSC (Δ*H*_DSC_).

*v*/°Cmin^−1^	*T*_DSC_/°C	Δ*H*_DSC_/Jg^−1^
1	35.7	10
2	36.0	10
5	36.4	11
10	37.1	10
